# Antagonism of miR-21 Reverses Epithelial-Mesenchymal Transition and Cancer Stem Cell Phenotype through AKT/ERK1/2 Inactivation by Targeting PTEN

**DOI:** 10.1371/journal.pone.0039520

**Published:** 2012-06-25

**Authors:** Mingli Han, Manran Liu, Yimeng Wang, Xin Chen, Jianli Xu, Yan Sun, Liuyang Zhao, Hongbo Qu, Yuanming Fan, Chengyi Wu

**Affiliations:** 1 Department of Endocrine Surgery, The First Affiliated Hospital of Chongqing Medical University, Chongqing, China; 2 The Key Laboratory of Laboratory Medical Diagnostics in the Ministry of Education and Department of Clinical Biochemistry, Chongqing Medical University, Chongqing, China; 3 Department of Emergency, The First Affiliated Hospital of Chongqing Medical University, Chongqing, China; 4 Department of General Surgery, The People’s Hospital of Jiangjin City, Chongqing, China; King Faisal Specialist Hospital & Research center, Saudi Arabia

## Abstract

**Background:**

Accumulating evidence suggested that epithelial-mesenchymal transition (EMT) and cancer stem cell (CSC) characteristics, both of which contribute to tumor invasion and metastasis, are interrelated with miR-21. MiR-21 is one of the important microRNAs associated with tumor progression and metastasis, but the molecular mechanisms underlying EMT and CSC phenotype during miR-21 contributes to migration and invasion of breast cancer cells remain to be elucidated.

**Methodology/Principal Findings:**

In this study, MDA-MB-231/anti-miR-21 cells were established by transfected hsa-miR-21 antagomir into breast cancer MDA-MB-231 cells. EMT was evaluated by the changes of mesenchymal cell markers (N-cadherin, Vimentin, and alpha-SMA), epithelial cell marker (E-cadherin), as well as capacities of cell migration and invasion; CSC phenotype was measured using the changes of CSC surface markers (ALDH1 and CD44), and the capacity of sphereforming (mammospheres). We found that antagonism of miR-21 reversed EMT and CSC phenotype, accompanied with PTEN up-regulation and AKT/ERK1/2 inactivation. Interestingly, down-regulation of PTEN by siPTEN suppressed the effects of miR-21 antagomir on EMT and CSC phenotype, confirming that PTEN is a target of miR-21 in reversing EMT and CSC phenotype. The inhibitors of PI3K-AKT and ERK1/2 pathways, LY294002 and U0126, both significantly suppressed EMT and CSC phenotype, indicating that AKT and ERK1/2 pathways are required for miR-21 mediating EMT and CSC phenotype.

**Conclusions/Significance:**

In conclusion, our results demonstrated that antagonism of miR-21 reverses EMT and CSC phenotype through targeting PTEN, via inactivation of AKT and ERK1/2 pathways, and showed a novel mechanism of which might relieve the malignant biological behaviors of breast cancer.

## Introduction

Epithelial-mesenchymal transition (EMT) is associated with increased aggressiveness and metastasis in carcinomas, including breast cancer, as it allows cells to migrate and invade surrounding issues and escape into the bloodstream, en route to establishing metastasis. Once these metastatic cells reach their destination, they can undergo mesenchymal- epithelial transition (MET) to establish secondary tumors, resulting in cancer spreading and treatment failure [1]–[3]. On the molecular level, cells undergoing EMT towards a more mesenchymal phenotype involves loss or lowered the expression of epithelial markers such as E-cadherin, and increased the expression of mesenchymal markers such as N-cadherin, Vimentin, and alpha-SMA [2], [4], [5]. There are studies suggested that EMT in breast cancer is tightly linked to the basal-like phenotype breast cancer subgroup and cancer stem cells (CSCs) [6]–[8].

CSCs are predicted to be critical drivers of tumor progression due to CSC characteristics including self-renewal capacity, limitless proliferative potential and metastasis potential, suggesting that targeting CSC characteristics would likely eliminate CSCs which are the “seeds” of tumor recurrence and metastasis. Although the CSC hypothesis suggests that tumors can arise from stem/progenitor cells, studies from many laboratories demonstrated that EMT can endow cells which possess CSC characteristics as well as a more motile invasive phenotype [7]–[10]. Previously studies have been confirmed that breast cancer contains a CSC-compartment [11]–[13], which can be enriched by purifying Aldehyde dehydrogenase 1 (ALDH1)-positive cells [13] or CD44^+^/CD24^−/low^ cells [11] by sorting, and also by purifying sphereforming cells (mammospheres) from parental cells [12]. There are studies demonstrated that EMT phenotype is highest in the CD44^+^/CD24^−/low^ breast cancer CSCs (7), and CSCs enriched from breast tumors and metastatic breast pleural effusions express markers similar to cells that have undergone an EMT [7], [14]. Similarly, EMT and CSC markers are also frequently associated with breast cancers that have a propensity to metastasis, such as basal-like phenotype breast cancer subgroup [15] and metaplastic [16] breast cancers.

MicroRNAs (miRNAs) are a family of small non-coding RNA molecules which regulate gene expression by base pairing to the 3′-UTR of the target mRNA. Recently, a series of miRNAs have been shown to play critical roles in the progression and metastasis of human malignancy [17], [18], including breast cancer. MiR-21 is one of the first miRNAs detected in the human genome, which also is one of miRNAs known to be up-regulated in all types of human malignancies [19]. Recent studies indicated that several tumor suppressors including phosphatase and tensin homolog deleted on chromosome ten (PTEN) [20], tumor suppressor gene tropomyosin 1 (TPM1) [21], programmed cell death 4 (PDCD4) [22], maspin [23], and matrix metalloproteinases inhibitors RECK and TIMP3 [24] were targets of miR-21, suggesting that miR-21 is an important oncogenic miRNA which is closely related to tumor growth and metastasis.

There are studies demonstrated that miR-21 is an important component of the cellular signaling circuitry that regulates the EMT program [25]–[27], as well as associates with CSC signatures [26], [28], suggesting that miR-21 plays a key role in the process of EMT and acquisition of CSC characteristics, which consistent with our previously study [29], but the underlying mechanisms remain unclear.

Recent studies have demonstrated that miR-21 increases tumor cell proliferation, migration, and invasion through targeting PTEN [20], a tumor suppressor gene that is an antagonist of phosphatidylinotidol 3-kinase (PI3K) by removing the 3′-UTR phosphate of phosphatidylinositol 3,4,5-trisphosphate (PIP3). But the role of PTEN in miR-21 inducing EMT and CSC phenotype remains to be elucidated. It is well known that PTEN negatively regulates the PI3K-protein kinase B (AKT) pathway [30]and mitogen-activated protein kinase (MAPK)/extracellular signal regulated kinase1/2 (ERK1/2) pathway [31], as well as both AKT and ERK1/2 pathways were considered to be relevant to the maintenance of EMT and CSC characteristics [32]–[36].

These results strongly suggest that miR-21 might via AKT and ERK1/2 pathways by targeting PTEN regulate EMT and CSC phenotype. In the present study, we have revealed that antagonism of miR-21reversed EMT consistent with CSC phenotype via AKT and ERK1/2 pathways by targeting PTEN, indicates a molecular pathway which might relieve the malignant biological behaviors of breast cancers.

## Results

### Transfected hsa-miR-21 Antagomir Decreased the Expression of miR-21 in MDA-MB-231 Cells

**Figure 1:**
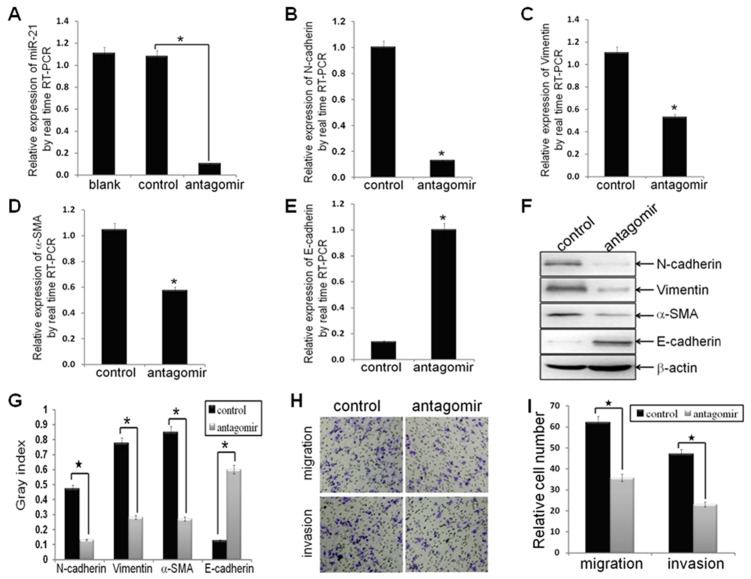
Antagonism of miR-21 reversed EMT phenotype, as well as decreased cell migration and invasion. MDA-MB-231 cells were transfected with hsa-miR-21 antagomir or hsa-miR-21 antagomir control at a final concentration of 50 nmol for 48 h. (A) MDA-MB-231 cells were treated with hsa-miR-21 antagomir decreased the expression of miR-21, as compared to control groups (n1 = n2 = 3; p = 0.0015), by real-time RT-PCR analysis. (B-E) The mRNA levels of mesenchymal biomarkers (N-cadherin, Vimentin and alpha-SMA) and epithelial biomarker (E-cadherin) in MDA-MB-231/anti-miR-21 cells and MDA-MB-231/control cells, as measured by real-time RT-PCR analysis. The real-time RT-PCR reactions were performed in a 20 µl reaction volume in triplicate, simultaneously. (F, G) The relative protein levels of EMT markers in indicated cells were shown by Western blot analysis, and bands were semi-quantified using ImageJ software. Beta-actin was used as loading control. (H, I) The migratory and invasive properties of indicated cells were tested in migration and invasion assay in Transwell inserts. Penetrated cells were counted and analyzed in histogram. Data represent at least three experiments done in triplicate. (*indicates p<0.05; ^★^ indicates p<0.001).

To explore whether hsa-miR-21 antagomir can decrease the expression of miR-21, hsa-miR-21 antagomir or its negative control was transfected into MDA-MB-231 cells for 48 h, then the relative expression of miR-21 was measured by real time RT-PCR. The relative expression of miR-21 was 0.10711±0.01272 in MDA-MB-231/anti-miR-21 cells, which was significantly down-regulated, as compare to 1.07862±0.09722 in negative control groups (p = 0.0015; Fig. 1A). The results suggested that hsa-miR-21 antagomir could decrease the expression of miR-21 in MDA-MB-231 cells.

### Antagonism of miR-21 Reversed EMT, as well as Decreased Migration and Invasion in MDA-MB-231 Cells

To investigate the effects of forced antagonism of miR-21 on the EMT process of MDA-MB-231 cells, the relative mRNA and protein expression of mesenchymal phenotype cell biomarkers (N-cadherin, Vimentin, and alpha-SMA), and epithelial phenotype cell biomarker (E-cadherin) in MDA-MB-231/anti-miR-21 cells and corresponding control cells were measured. Antagonism of miR-21 decreased the relative mRNA levels of N-cadherin, Vimentin, and alpha-SMA (p = 0.0038, p = 0.0018, and p = 0.0023, respectively; Fig. 1B, C, D), while increased the relative mRNA level of E-cadherin (p = 0.0017; Fig. 1E) in MDA-MB-231/anti-miR-21 cells, as compared to corresponding control cells, by real time RT-PCR analysis. The results suggested that antagonism of miR-21 could decrease the mRNA expression of mesenchymal phenotype cell biomarkers, while increase the mRNA expression of epithelial phenotype cell biomarker. The results from Western blot analysis demonstrated that the relative protein levels of N-cadherin, Vimentin, and alpha-SMA were down-regulated significantly (p = 0.0003, p = 0.0026, and p = 0.04, respectively; Fig. 1F, G), while that of E-cadherin was up-regulated significantly (p = 0.0053; Fig. 1F, G) in MDA-MB-231/anti-miR-21 cells, as compared to control cells. The results suggested that antagonism of miR-21 could decrease the protein expression of mesenchymal phenotype cell biomarkers, while increase that of epithelial phenotype cell biomarker. These results clearly demonstrated that antagonism of miR-21 could reverse the EMT, relying on inhibit EMT phenotypic biomarkers. Functionally, the relative migrated and invaded cell numbers of MDA-MB-231/anti-miR-21 cells were significantly inferior to negative control (p<0.0001, p<0.0001, respectively; Fig. 1H, I), suggested that antagonism of miR-21 could reduce cell migration and invasion in MDA-MB-231 cells, which further emphasized the function of miR-21 in the invasion and metastasis of breast cancer cells.

### Antagonism of miR-21 Reversed CSC Phenotype in MDA-MB-231 Cells

**Figure 2:**
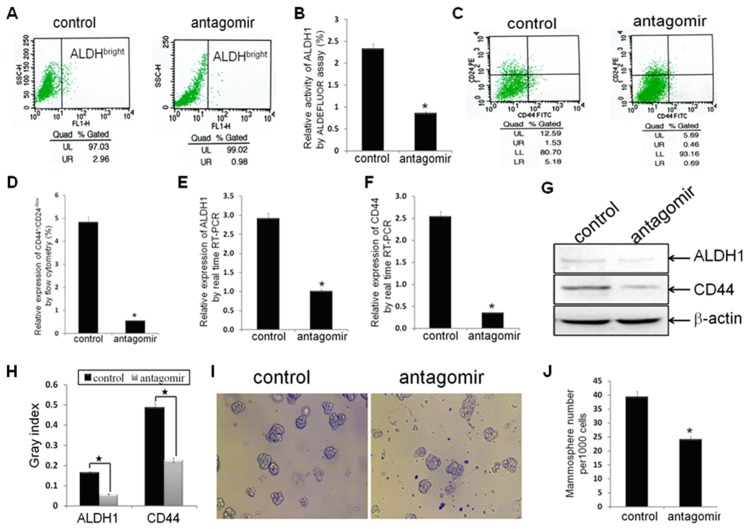
Antagonism of miR-21 reversed CSC phenotype. (A, B) ALDH1 enzymatic activity (ALDH^bright^) in established breast cancer MDA-MB-231/anti-miR-21 cells and MDA-MB-231/control cells (n1 = n2 = 3) were detected using the ALDEFLUOR assay. (C, D) The CD44^+^/CD24^−/low^ phenotype in indicated cells (n1 = n2 = 3) were detected by FACS analysis. (E, F) The relative mRNA levels of ALDH1 and CD44 were detected by real time RT-PCR assay. (G, H) The relative protein levels of ALDH1 and CD44 were detected by Western blot analysis. Beta-actin was used as loading control. (I, J) The number of mammospheres from 1000 MDA-MB-231/anti-miR-21 cells or MDA-MB-231/control cells was counted under microscope. All the data represent at least three experiments done in triplicate. (*indicates p<0.05; ^★^ indicates p<0.001).

To examine the effects of antagonism of miR-21 on CSC phenotype in MDA-MB-231 cells, the proportion of cells with ALDH1^+^ (ALDH^bright^) and CD44^+^/CD24^−/low^ in MDA-MB-231/anti-miR-21 cells and negative control cells were measured. The percentage of cells with ALDH1^+^ in MDA-MB-231/anti-miR-21 cells was 0.85*±*0.092, significantly less than 2.323±0.315 in negative control groups (p = 0.0051; Fig. 2A, B), by ALDEFLUOR assay; the percentage of cells with CD44^+^/CD24^−/low^ in MDA-MB-231/anti-miR-21cells was 0.543±0.129, also significantly lower than 4.807±1.062 in control groups (p = 0.0094; Fig. 2C, D), by FACS analysis. These results suggested that antagonism of miR-21 could decrease the breast cancer CSC proportion, which expressing CSC surface biomarkers ALDH1^+^ and CD44^+^/CD24^−/low^. Moreover, the relative mRNA and protein expression of ALDH1 and CD44 in MDA-MB-231/anti-miR-21 cells and control cells were also measured. The relative mRNA levels of ALDH1 and CD44 in MDA-MB-231/anti-miR-21 cells were significantly decreased as compared to control groups (p = 0.0039, p = 0.0034, respectively; Fig. 2E, F), as assessed by real time RT-PCR analysis, which suggested that antagonism of miR-21 could decrease the mRNA expression of ALDH1 and CD44. The protein expression of ALDH1 and CD44 were also decreased accordingly (p = 0.0002, p = 0.0005, respectively; Fig. 2G, H), by Western blot analysis, suggested that antagonism of miR-21 could decrease the protein expression of ALDH1 and CD44. These results indicated that antagonism of miR-21 could reverse CSC phenotype in MDA-MB-231 cells, which further suggested that miR-21 could involve in the regulation of CSC characteristics. More interestingly, the mammosphere number in MDA-MB-231/anti-miR-21 cells was significantly inferior to control groups (p = 0.0017; Fig. 2I, J), indicated that antagonism of miR-21 could decrease formation of mammospheres, which further suggested that miR-21 could regulate CSC characteristics, including the capacities of self-renewal and clonogenicity.

### Antagonism of miR-21 Induced Over-expression of PTEN

**Figure 3:**
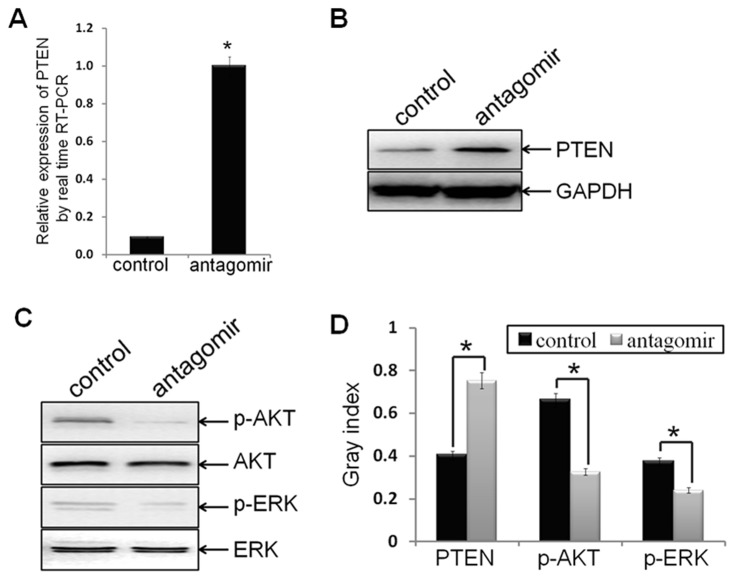
Antagonism of miR-21 induced the expression of PTEN, as well as inactivated AKT and ERK1/2. (A) Ectopic expression of PTEN mRNA in MDA-MB-231/anti-miR-21 cells and MDA-MB-231/control cells were verified by real time RT-PCR assay (p = 0.003). (B, C, D) Protein levels of PTEN, p-AKT, AKT, p-ERK1/2, and ERK1/2 in indicated cells were detected by Western blot analysis, and bands were semi-quantified using ImageJ software. GAPDH was used as loading control. (*indicates p<0.05).

Recent studies have demonstrated that miR-21 increased cell proliferation, migration and invasion through modulating tumor suppressor gene PTEN [20], but the role of PTEN in miR-21 regulating tumor EMT and CSC phenotype remains to be elucidated. To explore whether antagonism of miR-21 regulate PTEN expression, the mRNA and protein levels of PTEN were measured, by real time RT-PCR analysis and Western blot assay, respectively. As expected, both the mRNA and protein expression of PTEN in MDA-MB-231/anti-miR-21 cells were strongly up-regulated, as compared to control groups (p = 0.003, p = 0.0437, respectively; Fig. 3A, B, D). These results demonstrated that antagonism of miR-21 could up-regulate the expression of PTEN.

### Antagonism of miR-21 Inactivated AKT and ERK1/2

AKT and ERK1/2 are two major signaling pathways in regulating cell proliferation, migration and survival, and both also were regulated by PTEN, but the roles of AKT and ERK1/2 pathways in miR-21 regulating tumor EMT and CSC phenotype remains to be elucidated. To determine the signaling molecules that are involving in antagonism of miR-21 reversing EMT and CSC phenotype, the protein levels of phosphorylated AKT (p-AKT) and AKT, phosphorylated ERK1/2 (p-ERK1/2) and ERK1/2 were measured by Western blot analysis. The protein levels of p-AKT and p-ERK1/2 in MDA-MB-231/anti-miR-21 cells were strongly decreased as compared to control groups (p = 0.0173, p = 0.0126, respectively; Fig. 3C, D), confirmed that antagonism of miR-21 could suppress AKT and ERK1/2 activation in process of reversing EMT and CSC phenotype.

### Re-expression of miR-21 Induced EMT and CSC Phenotype, Accompanied with Down-expression of PTEN, as well as Activation of AKT and ERK1/2

**Figure 4:**
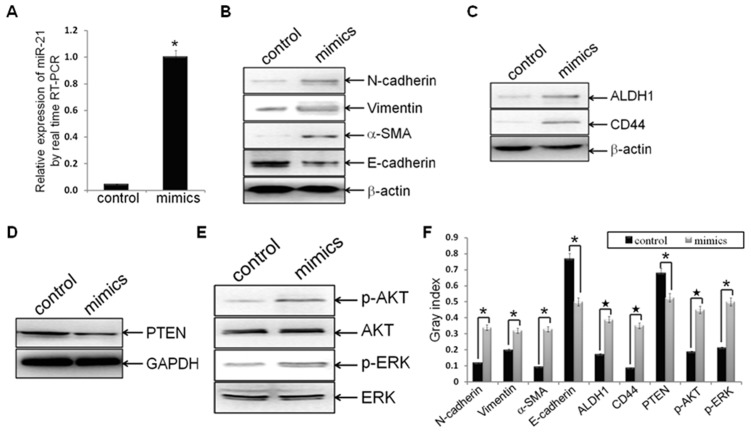
Hsa-miR-21 mimics induced EMT and CSC phenotype, accompanied with PTEN down-regulation and AKT/ERK1/2 activation. Established MDA-MB-231/anti-miR-21 cells were transfected with hsa-miR-21 mimics at a concentration of 40 nmol for 72 h. (A) MDA-MB-231/anti-miR-21 cells were treated with hsa-miR-21 mimics elevated the expression of miR-21, as compared to control groups (n1 = n2 = 3; p = 0.00373), by real-time RT-PCR analysis. (B-F) Protein levels of mesenchymal markers (N-cadherin, Vimentin and alpha-SMA) (B), epithelial marker (E-cadherin) (B), CSC markers (ALDH1 and CD44) (C), PTEN (D), p-AKT and AKT (E), as well as p-ERK1/2 and ERK1/2 (E) in indicated cells were measured by Western blot analysis, and bands were semi-quantified using ImageJ software (F). Beta-actin or GAPDH was used as loading control. (*indicates p<0.05; ^★^ indicates p<0.001).

To further confirm the role of miR-21 in regulating tumor EMT and CSC phenotype, hsa-miR-21 mimics or mimics negative control was transfected into established MDA-MB-231/anti-miR-21 cells. The expression of miR-21 increased to more than 22-fold after hsa-miR-21 mimics treated, as compared to the negative control (p = 0.0037; Fig. 4A), indicated that hsa-miR-21 mimics transfection could increase the relative expression of miR-21.

To examine whether forced re-expression of miR-21 can induce EMT and CSC phenotype, down-regulate expression of PTEN, as well as activate AKT and ERK1/2 pathways, the protein expression of EMT biomarkers (N-cadherin, Vimentin, alpha-SMA, and E-cadherin), CSC markers (ALDH1 and CD44), PTEN, p-AKT, AKT, p-ERK1/2, and ERK1/2 were measured by Western blot assay. As compared to the negative control groups, forced re-expression of miR-21 increased the protein expression of N-cadherin, Vimentin, alpha-SMA, ALDH1 and CD44 (p = 0.0136, p = 0.0397, p = 0.0067, p = 0.0002, and p = 0.0006, respectively; Fig. 4B, C, F), while decreased the expression of E-cadherin (p = 0.0014; Fig. 4B, F), suggested that re-expression of miR-21 could induce EMT and CSC phenotype in established MDA-MB-231/anti-miR-21 cells. Meanwhile, re-expression of miR-21 decreased the expression of PTEN (p = 0.0081; Fig. 4D, F), demonstrated that re-expression of miR-21 could affect the expression of miR-21 direct target in the cells. Furthermore, re-expression of miR-21 also increased the expression of p-AKT and p-ERK1/2 in the cells (p = 0.0008, p = 0.0022, respectively; Fig. 4E, F), indicated that re-expression of miR-21 could activate AKT and ERK1/2 pathways. These results supported that miR-21 could regulate EMT and CSC phenotype, and accompany with alterations of PTEN and AKT/ERK1/2 pathways.

### MiR-21 Targeted PTEN in Reversing EMT and CSC Phenotype

**Figure 5:**
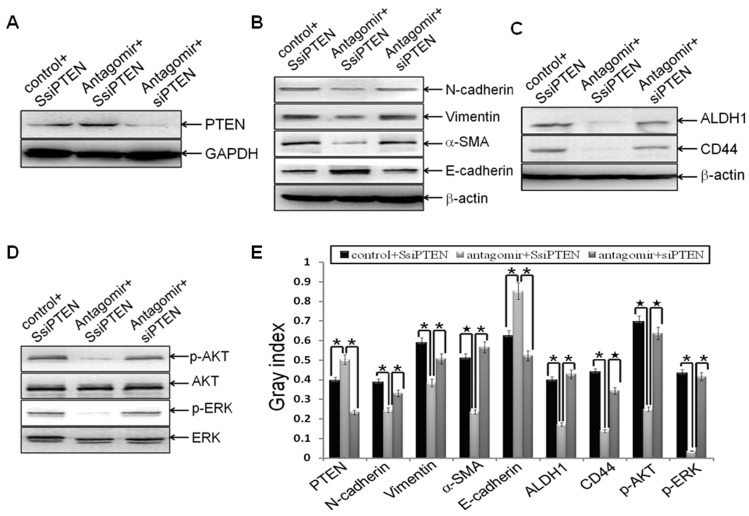
PTEN was the downstream target of miR-21 during reversing EMT and CSC phenotype . MDA-MB-231 cells were transfected with siPTEN or the scrambled control SsiPTEN at a final concentration of 50 nmol for 24 h. Then the cells were transfected with hsa-miR-21 antagomir or negative control at a final concentration of 50 nmol for 72 h. Cells were trypsinized, and the relative protein levels of PTEN (A), EMT markers (B), CSC surface markers (C), p-AKT and AKT (D), as well as p-ERK and ERK (D) were measured and semi-quantified (E) as stated before. The representative plugs from treatments of SsiPTEN plus miR-21 antagomir control, SsiPTEN plus miR-21 antagomir, and siPTEN plus miR-21 antagomir were shown in the picture. Beta-actin or GAPDH was used as loading control. (*indicates p<0.05; ^★^ indicates p<0.001).

To determine whether depletion of PTEN can block miR-21 antagomir reversing EMT and CSC phenotype, siPTEN or the control SsiPTEN was transfected into MDA-MB-231 cells. After 24 h, the cells were transfected with miR-21 antagomir or negative control, and then EMT and CSC phenotype were examined as described above. We found that siPTEN significant down-regulated the expression of PTEN (p = 0.002; Fig. 5A, E), as compared to SsiPTEN groups. Consistent with the effects of miR-21 antagomir on EMT and CSC phenotype in MDA-MB-231 cells (Fig. 1 and Fig. 2), antagonism of miR-21 also reversed EMT (N-cadherin, p = 0.0025; Vimentin, p = 0.0025; alpha-SMA, p = 0.0003; E-cadherin, p = 0.0308; Fig. 5B, E) and CSC phenotype (ALDH1, p = 0.0014; CD44, p = 0.002; Fig. 5C, E) in MDA-MB-231/SsiPTEN cells (*SsiPTEN+miRNA antagomir negative control groups vs. SsiPTEN+miR-21 antagomir groups*); depletion of PTEN by siPTEN blocked miR-21-antagomir-reversing EMT (N-cadherin, p = 0.0172; Vimentin, p = 0.0113; alpha-SMA, p = 0.0015; E-cadherin, p = 0.016; Fig. 5B, E) and CSC phenotype (ALDH1, p = 0.0042; CD44, p = 0.0004; Fig. 5C, E) (*SsiPTEN + miR-21 antagomir groups vs. siPTEN + miR-21 antagomir groups*). These results suggested that depletion of PTEN could block miR-21-antagomir -reversing EMT and CSC phenotype, which further suggested that antagonism of miR-21 could target PTEN to reversing EMT and CSC phenotype. Moreover, depletion of PTEN activated miR-21-antagomir-blocking AKT and ERK1/2 pathways (p-AKT, p<0.0001; p-ERK1/2, p = 0.0037; Fig. 5D, E) (*SsiPTEN + miR-21 antagomir groups vs. siPTEN + miR-21 antagomir groups*), indicated that AKT and ERK1/2 pathways are downstream pathways of PTEN in regulating EMT and CSC phenotype.

### MiR-21 Regulated EMT and CSC Phenotype, as well as Cell Proliferation through AKT and ERK1/2 Pathways

**Figure 6:**
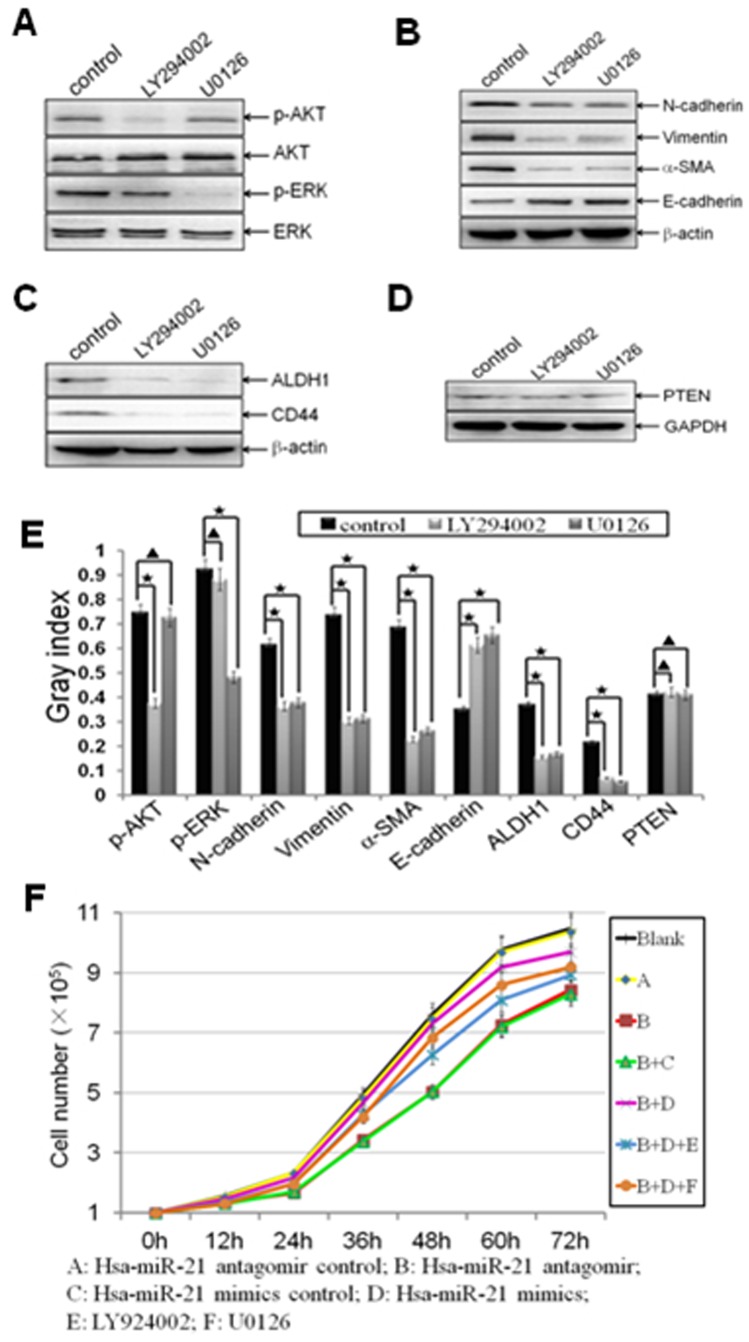
MiR-21 regulated EMT and CSC phenotype through mediating AKT and ERK1/2 activation. Established MDA-MB-231/anti-miR-21 cells were transfected with hsa-miR-21 mimics at a concentration of 40 nmol for 72 h. Then the cells were trypsinized, and treated with LY294002 (20 µmol/l) or U0126 (10 µmol/l) for 24 h. (A-E) The relative protein levels of p-AKT and AKT (A), p-ERK and ERK (A), EMT markers (B), CSC surface markers (C), as well as PTEN (D) from blank control, LY294002, and U0126 treatment groups were shown, and semi-quantified (E) as stated before. Beta-actin or GAPDH was used as loading control. (*indicates p<0.05; ^★^ indicates p<0.001; ^▴^ indicates p>0.05). (F) The effects of antagonism of miR-21, re-expression of miR-21, LY294002, and U0126 on cell proliferation by cell count (A *vs.* B, p = 0.0077; B+C *vs.* B+D, p = 0.0091; B+D *vs.* B+D+E, p = 0.0109; B+D *vs.* B+D+F, p = 0.0031).

To further investigate the roles of AKT and ERK1/2 pathways in miR-21 regulating EMT and CSC phenotype, the MDA-MB-231/anti-miR-21 cells were transfected with miR-21 mimics at a concentration of 40 nmol for 72 h, and then the cells were treated with LY294002 (the inhibitor of PI3K-AKT) at 20 µmol/l or U0126 (the inhibitor of ERK1/2) at 10 µmol/l for 24 h. We confirmed that LY294002 treatment blocked re-expression of miR-21 inducing AKT activation (p<0.0001; Fig. 6A, E), while U0126 treatment abolished re-expression of miR-21 inducing ERK1/2 activation (p<0.0001; Fig. 6A, E). More interestingly, re-expression of miR-21 inducing EMT and CSC phenotype were inhibited by LY294002 (N-cadherin, p = 0.0005; Vimentin, p = 0.0005; alpha-SMA, p = 0.0008; E-cadherin, p = 0.0002; ALDH1, p<0.0001; CD44, p<0.0001; Fig. 6B, C, E) or U0126 (N-cadherin, p<0.0001; Vimentin, p = 0.0005; alpha-SMA, p = 0.0004; E-cadherin, p = 0.0003; ALDH1, p<0.0001; CD44, p = 0.0005; Fig. 6B, C, E) in the cells, indicated that both AKT and ERK1/2 pathways are requiring for miR-21 in inducing EMT and CSC phenotype, which further suggested that AKT and ERK1/2 pathways are two parallel downstream pathways of miR-21 for regulating EMT and CSC phenotype. Furthermore, both LY294002 and U0126 have no proven effect on PTEN (p = 0.1208, p = 0.4361, respectively; Fig. 6D, E), which also supported that AKT and ERK1/2 pathways are downstream pathways of PTEN.

Besides, to verify the roles of AKT and ERK1/2 pathways in miR-21 regulating cell proliferation in the cells that mentioned above, the cell growth numbers of the cells were calculated at 0, 12, 24, 36, 48, 60 and 72 h after treatments by cell count. We found that forced antagonism of miR-21 decreased cell proliferation in MDA-MB-231 cells (p = 0.0077; Fig. 6F), while re-expression of miR-21 elevated cell proliferation in MDA-MB-231/anti-miR-21 cells, during 0–72 h (p = 0.0091; Fig. 6F). The results suggested that miR-21 could regulate the cell propagation to a certain degree in MDA-MB-231 cells. Meanwhile, both AKT and ERK1/2 inhibitions were decreased cell proliferation (p = 0.0109, p = 0.0031, respectively; Fig. 6F), which suggested that the regulation of AKT or ERK1/2 linked to changes in cell proliferation.

## Discussion

MiRNAs are noncoding small RNAs that may act as oncogenes or tumor suppressor genes [37], [38]. Growing evidence showed that miR-21 over-expression is detected in various kinds of human cancers including breast cancer, and is associated with EMT [25]–[27] and CSC characteristics [26], [28]. But the direct roles and central molecular mechanisms of miR-21 in regulating breast cancer EMT and CSC phenotype remains to be elucidated. In this study, we found that antagonism of miR-21 in MDA-MB-231 cells reversed EMT and CSC phenotype, up-regulated the expression of PTEN, as well as inactivated AKT and ERK1/2 pathways. The results demonstrated that antagonism of miR-21 is sufficient to reverse EMT and CSC phenotype through modulating the expression of PTEN and AKT/ERK1/2 pathways, providing some direct evidence and molecular mechanisms that miR-21 is able to regulate EMT and CSC phenotype.

To investigate the role of miR-21 in regulating EMT and CSC phenotype of breast cancer cells, antagomir of miR-21, which can specifically bind to and inhibit endogenous miR-21 molecules, or its negative control oligomer, was transfected into MDA-MB-231 cells. The antagomir decreased 90% of endogenous miR-21 expression in the cells (Fig. 1A). Interestingly, the relative EMT and CSC phenotype were reversed by antagonism of miR-21(Fig. 1B–G; Fig. 2A–J), which consistent with miR-21 involves in promoting EMT and (or) CSC phenotype in pancreatic cancer cells [26], [28], thyroid carcinoma cells [27], and our previously study in MCF-7 cells [29]. Accompanied with the results, antagonism of miR-21 significantly increased the expression of PTEN (Fig. 3A, B, D), as well as decreased AKT and ERK1/2 activation (Fig. 3C, D). These results demonstrated that miR-21 plays an important role in mediating EMT and CSC phenotype, accompany by regulating the expression of PTEN, as well as AKT and ERK1/2 activation.

Next, we explored the underlying mechanisms and relative signaling molecules in antagonism of miR-21 reversing EMT and CSC phenotype. In consistent with that miR-21 suppresses the function of tumor suppressor PTEN expression by binding its 3′-UTR [20], we proved that antagonism of miR-21 up-regulated PTEN expression (Fig. 3A, B, D). As expected, PTEN down-expression notably blocked miR-21-reversing EMT and CSC phenotype (Fig. 5B, C, E), confirmed that antagonism of miR-21 exhibits its role partly by promoting PTEN expression, and the recovery of PTEN expression would reduce its function of tumor suppressor.

Furthermore, we also found that re-expression of miR-21 by miR-21 mimics led to the activation of AKT and ERK1/2 pathways (Fig. 4E, F). More interestingly, treated the cells with PI3K-AKT or ERK1/2 inhibitor (LY294002 or U0126), prohibited miR-21 mimics regaining EMT and CSC phenotype (Fig. 6B, C, E). The results suggested that the effects of AKT and (or) ERK1/2 on miR-21-regulating EMT and CSC phenotype, in accordance with that AKT and (or) ERK1/2 pathways are required for promote EMT and (or) CSC phenotype in breast cancer MCF-7 cells [32], breast epithelial cells [33], [34], colon cancer cells [35], cervical cancer cells [32], and ovarian carcinomas cells [36].

**Figure 7:**
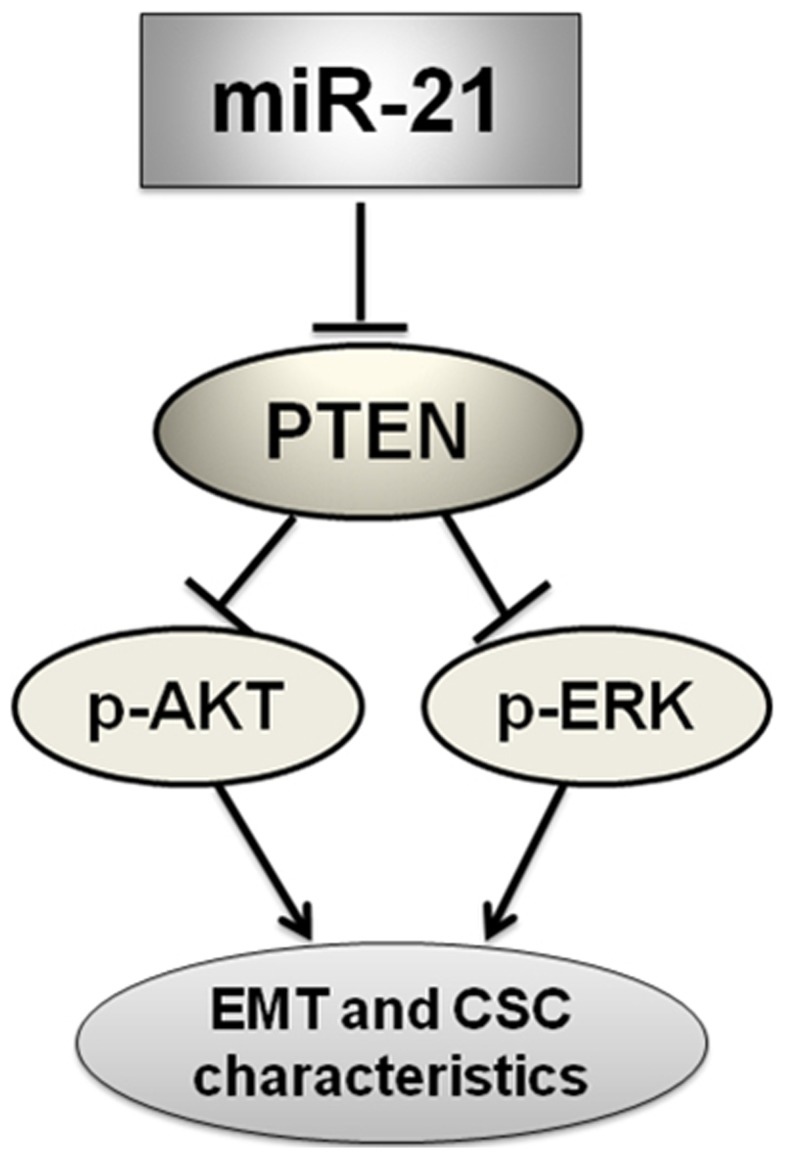
Schematic diagram of the proposed model: possible mechanism of miR-21 regulates EMT and CSC phenotype. Antagonism of miR-21 could inactivate AKT and ERK1/2 pathways presumably through PTEN up-regulation, and finally reverse EMT and CSC phenotype in MDA-MB-231 cells.

In summary, our results demonstrated that antagonism of miR-21 reverses EMT and CSC phenotype through AKT and ERK1/2 pathways inactivation by inducing PTEN expression in MDA-MB-231 cells (Fig. 7). This study showed a direct role and novel mechanism of miR-21 in inversing EMT and CSC phenotype, and the information may be useful to develop a new therapy for treatment of breast cancers in the future.

## Materials and Methods

### Reagents and Antibodies

Human has-miR-21 (MIMAT0000076) antagomir and negative control, mimics and negative control were purchased from Ribobio (Guangzhou, China). The PCR primers were synthesized by Sangon Biotech (Shanghai, China). Real time RT-PCR assay kits were purchased from Takara (Dalian, China). Specific siPTEN duplexes and scramble control siRNA sequence (SsiPTEN) were purchased from Ribobio. LY294002 and U0126 were purchased from Sigma (St. Louis, MO, USA). Lipofectamine™2000 was purchased from Invitrogen (Carlsbad, CA, USA). ALDEFLUOR® Kit was purchased from Aldagen (Durham, NC, USA). Fluorescein isothiocyanate (FITC)-labeled anti-CD44 antibody and phycoerythrin (PE)-labeled anti-CD24 antibody were purchased from BioLegend (San Diego, CA, USA). Primary antibodies including rabbit antihuman E-cadherin antibody (No: BS1098; Bioworlde, St. Louis, MO, USA), N-cadherin antibody (No: BS2224; Bioworlde), Vimentin antibody (No: BS1776; Bioworlde), alpha-SMA antibody (No: ab5694; Abcam, Cambridge, UK), ALDH1 antibody (No: ab51028; Abcam), CD44 antibody (No: BA0321; Boster, Wuhan, China), PTEN antibody (No: BS1304; Bioworlde), AKT antibody (No: BS1810; Bioworlde), phospho-AKT (p-AKT) antibody (No: BS4006; Bioworlde), ERK1/2 antibody (No: BS3628; Bioworlde), phospho-ERK1/2 (p-ERK1/2) antibody(No: BS4621; Bioworlde), GAPDH antibody (No: AP0063; Bioworlde) and beta-actin antibody (No: BA2305; Boster), and HRP-conjugated anti-rabbit IgG secondary antibody (No: BA1055; Boster) were used for Western blot analysis.

### Cell Culture

Human breast cancer MDA-MB-231 cells were maintained in high-glucose DMEM supplemented with 5% FBS (Gibico, Grand Island, NY, USA), 100 units/ml penicillin, 100 µg/ml streptomycin and 1% glutamine (Invitrogen). Cells were grown at 37°C in an atmosphere containing 5% CO_2_.

### Antagonism and Re-expression of miR-21

5×10^5^ cells were seeded in six-well plates and grown to 60% confluence. Human has-miR-21 (MIMAT0000076) antagomir or its negative control was directly transfected into MDA-MB-231 cells at a final concentration of 50 nmol/l, according to the manufacturer’s protocol. Human has-miR-21 (MIMAT0000076) mimics or its negative control was allowed to form transfection complexes with Lipofectamine™2000 in free of serum Opti-MEM® I (Invitrogen) at a final concentration of 40 nmol/l, according to the manufacturer’s protocol.

### Real Time RT-PCR Analysis

For miR-21, the miR-21 reverse transcription (RT) primer (5′-GTCGTATCCAGTGCAGGGTCCGAGGTATTCGCACTGGATACGACTCAACA-3′) and real time RT-PCR primers (Forward: 5′-GCCCGCTAGCTTATCAGACTGATG-3′; Reverse: 5′-GTGCAGGGTCCGAGGT-3′) were synthesized by Sangon Biotech. MiRNAs was extracted from cells using a mirVana miRNA Isolation Kit (Applied Biosystems, Foster City, CA, USA). SYBR Green–based real time RT-PCR was performed using SYBR® PrimeScript® miRNA RT-PCR Kit (Takara) to measure the expression of mature miR-21 in cells by a MiniOpticon™ Two-Color Real-Time PCR Detection System (Bio-Rad, Hercules, CA, USA). U6 was used as endogenous control.

For mRNAs, total RNA from cells was isolated using TRIzol reagent (Invitrogen). Real time RT-PCR reactions were carried out using SYBR® Premix Ex Taq™ II (Takara). Beta-actin was used as endogenous control.

### Western Blot Analysis

Western blot analysis was conducted as our previously study [29], [39]. Briefly, the protein was separated by SDS-PAGE (8%, 10%, or 12%) and transferred to PVDF membranes. Nonspecific binding sites were blocked by incubating with TBST containing 5% (w/v) non-fat dried milk. Then incubated with primary antibodies (as described above) and HRP-conjugated anti-rabbit IgG secondary antibody in order, and visualized by ECL chemiluminescence. Beta-actin or GAPDH was used as loading control. The bands were semi-quantified using ImageJ software.

### Migration and Invasion Assays

Cell migration and invasion assays were performed as our previously described [29], [40]. For invasion assay, 2.5×10^4^ cells were seeded on an 8-µm pore size Transwell insert (Corning Inc. Corning, NY, USA) coated with ECM (1∶7.5) (Sigma), while cell migration assay did not coat with ECM. After 24 h of incubation at 37°C in an atmosphere containing 5% CO_2_, the cells adherent to the upper surface of the filter were removed using a cotton applicator. Then cells were stained with 0.4% crystal violet dissolved in methanol, and the numbers of cells on the bottom were counted.

### ALDEFLUOR Assay

The ALDEFLUOR kit was used to isolate the population with high ALDH1enzymatic activity (ALDH^bright^) as previously described [13], [29]. Briefly, cells were incubated in ALDEFLUOR assay buffer containing ALDH substrate bodipy-aminoacetaldehyde (BAAA, 1 µmol/l). In each experiment, a sample of cells was stained under identical conditions with diethylaminobenzaldehyde (DEAB), a specific ALDH inhibitor, as negative control. Flow cytometry (FACS) analysis was used to measure ALDH^bright^ cell subpopulation.

### FACS of CD44^+^/CD24^−/low^ Cell Subpopulation

FACS of CD44^+^/CD24^−/low^ cell subpopulation was performed as our previously described [29], [40]. Briefly, at least 1×10^5^ unfixed cells were incubated with FITC-labeled anti-CD44 antibody and PE-labeled anti-CD24 antibody at 4°C in darkness for 30 min. Then the cells were analyzed by a FACScalibur flow cytometer (BD Biosciences, Franklin Lakes, NJ, USA) using CellQuest software.

### Mammosphere Formation Assay

Mammosphere formation assay was performed as described previously [12], [13], [29]. Density of 5×10^3^–1×10^4^ cells per milliliter single-cell suspensions were seed into six-well non-adherent plates in serum-free DMEM/F12 (Gibico) supplemented with 2% B27 (Invitrogen), 20 ng/ml EGF (PeproTech, London, UK), 20 ng/ml bFGF (PeproTech), 0.4% BSA (Sigma), and 5 µg/ml insulin. After 7 day of culture, the numbers of mammospheres were counted.

### Construction of siPTEN

Based on human PTEN mRNA sequence (NM_000314), specific siRNA duplexes were designed, synthesized and annealed by Ribobio. The selected RNA duplex (siPTEN) corresponding to nucleotides 1567–1585 of PTEN mRNA is defined as sense: 5′-GCUACCUGUUAAAGAAUCAdTdT-3′ and antisense: 5′-UGAUUCUUUAACAGGUAGCdTdT-3′. The scramble control siRNA sequence (SsiPTEN) was also designed by Ribobio, and has no significant homology to any known human gene sequence.

### Transfection of siPTEN

5×10^5^ cells were seeded in six-well plates and grown to 70% confluence. Human siPTEN or SsiPTEN was allowed to form transfection complexes with Lipofectamine™2000 (Invitrogen) in free of serum Opti-MEM® I (Invitrogen) at a final concentration of 50 nmol/l, according to the manufacturer’s protocol.

### Cell Proliferation Assay

Various cells were cultured in 10% FBS culture medium as mentioned in Cell Culture part. The cell growth numbers of the cells were calculated and analyzed by cell count at 0, 12, 24, 36, 48, 60 and 72 h after treatments.

### Statistical Analysis

All data were expressed as mean ± SD and were calculated by using statistics analysis software SPSS 13.0. Statistical difference of each treatment was compared by Student’s *t* test. The *P* value equal to or less than 0.05 was considered as statistical significance.
